# Effect of Thermospermine on the Growth and Expression of Polyamine-Related Genes in Rice Seedlings

**DOI:** 10.3390/plants8080269

**Published:** 2019-08-06

**Authors:** Minaho Miyamoto, Satoshi Shimao, Wurina Tong, Hiroyasu Motose, Taku Takahashi

**Affiliations:** Graduate School of Natural Science and Technology, Okayama University, Okayama 700-8530, Japan

**Keywords:** Arabidopsis, phloem, rice, spermine, thermospermine, xylem

## Abstract

A mutant defective in the biosynthesis of thermospermine, *acaulis5* (*acl5*), shows a dwarf phenotype with excess xylem vessels in *Arabidopsis thaliana*. Exogenous supply of thermospermine remarkably represses xylem differentiation in the root of seedlings, indicating the role of thermospermine in proper repression of xylem differentiation. However, the effect of thermospermine has rarely been investigated in other plant species. In this paper, we examined its effect on the growth and gene expression in rice seedlings. When grown with thermospermine, rice seedlings had no clearly enlarged metaxylem vessels in the root. Expression of *OsACL5* was reduced in response to thermospermine, suggesting a negative feedback control of thermospermine biosynthesis like in Arabidopsis. Unlike Arabidopsis, however, rice showed up-regulation of phloem-expressed genes, *OsHB5* and *OsYSL16*, by one-day treatment with thermospermine. Furthermore, expression of *OsPAO2* and *OsPAO6*, encoding extracellular polyamine oxidase whose orthologs are not present in Arabidopsis, was induced by both thermospermine and spermine. These results suggest that thermospermine affects the expression of a subset of genes in rice different from those affected in Arabidopsis.

## 1. Introduction

Thermospermine synthase is encoded by the *ACAULIS5* (*ACL5*) gene in *Arabidopsis thaliana* [1] and its loss-of-function mutant *acl5* shows a stunted growth along with excess xylem vessels [2,3,4,5]. The point-mutated *acl5* mRNA level is increased in *acl5* but is reduced by exogenous thermospermine [6], suggesting a negative feedback control of thermospermine biosynthesis. Previous studies have revealed that thermospermine plays a role in the repression of xylem differentiation through enhancing mRNA translation of *SAC51*, which encodes a basic helix-loop-helix (bHLH) protein [6,7]. The *SAC51* mRNA contains conserved upstream open-reading-frames (uORFs) in the 5’ leader region [8] and thermospermine functions in alleviating the inhibitory effect of the uORFs on the main ORF translation, although its precise mode of action remains unclear [9,10]. Expressions of *ACL5* and a member of the *SAC51* family, *SACL3*, are directly activated by bHLH heterodimers LHW-TMO5 and LHW-T5L1 in xylem precursor cells in the root [11] and these heterodimers play a key role in auxin-induced xylem formation [12]. Because SAC51 and SACL3 in turn compete with TMO5 or T5L1 to heterodimerize with LHW [11,13], thermospermine appears to be a part of the negative feedback regulation of auxin-induced xylem formation and thermospermine biosynthesis [14,15,16]. These results have been obtained solely from studies in Arabidopsis and, except for a study that reported on the negative feedback regulation of thermospermine homeostasis in poplar xylem tissues [17] and that showed the dwarf phenotype of cotton plants by silencing of *GhACL5* [18], the role of thermospermine in plant development has rarely been investigated in other plant species so far.

**Figure 1:**
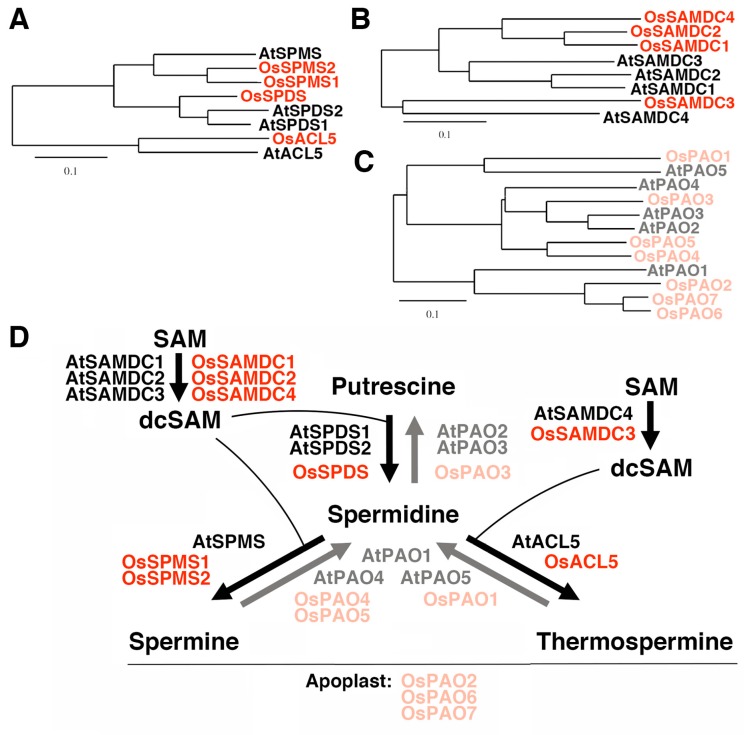
Polyamine biosynthetic and catabolic genes in Arabidopsis and rice. (**A**) Phylogenetic tree of spermidine synthase (SPDS), spermine synthase (SPMS), and thermospermine synthase (ACL5) isoforms. (**B**) Phylogenetic tree of *S*-adenosylmethionine decarboxylase (SAMDC) isoforms. (**C**) Phylogenetic tree of polyamine oxidase (PAO) isoforms. All trees based on the deduced amino acid sequences were constructed using ClustalW with the neighbor-joining method in the DNA Data Bank of Japan (DDBJ) website. The scale bar indicates the number of amino acid substitutions per site. (**D**) Pathways of polyamine biosynthesis and catabolism and the name of gene products mediating each reaction. Arabidopsis proteins are shown in black and light gray and rice proteins are in red and light red.

On the other hand, genes involved in polyamine biosynthesis and catabolism have been increasingly characterized in rice. Phylogenetic relationships based on recent studies together with the information of rice and Arabidopsis genome databases reveal that the rice genome has one gene for spermidine synthase, *OsSPDS*, one gene for thermospermine synthase, *OsACL5*, and two genes for spermine synthase, *OsSPMS1* and *OsSPMS2* (Figure 1A). Six putative genes for *S*-adenosylmethinine decarboxylase (SAMDC/AdoMetDC) are present in rice [19,20,21], among which two encode very short deduced polypeptides and may represent pseudo-genes. In Arabidopsis, *SAMDC4* has been suggested to be tightly involved in the biosynthesis of thermospermine because its expression is limited to vasculature and decreased by thermospermine and its loss-of-function mutant, *bud2*, shows a dwarf phenotype similar to that of *acl5* [22,23]. The orthologous gene in rice may be *OsSAMDC3* (Figure 1B). There are seven genes for polyamine oxidase (PAO) in rice [24,25,26]. *OsPAO2*, *OsPAO6*, and *OsPAO7*, may encode apoplastic enzymes catalyzing the terminal catabolism reaction [27] while their closest homolog in Arabidopsis, *AtPAO1*, encodes a cytoplasmic enzyme catalyzing the back-conversion of thermospermine or spermine to spermidine [28]. In Arabidopsis, *PAO5* is specifically expressed in vasculature and may be preferentially involved in the degradation of thermospermine [29,30,31,32]. The orthologous gene in rice appears to be *OsPAO1* (Figure 1C) [25,33]. According to the above-mentioned references and molecular phylogenetic trees, the pathways that are or may be catalyzed by these gene products in rice and Arabidopsis are summarized in Figure 1D. Expression of these rice genes, however, remains to be investigated in terms of the response to thermospermine.

Within this context, we here focus on the effect of thermospermine on the growth and expression of the genes related to polyamine biosynthesis, catabolism, and vascular development in rice as a model monocotyledonous species.

## 2. Results

### 2.1. Thermospermine Suppresses Xylem Vessel Expansion in the Root

**Figure 2:**
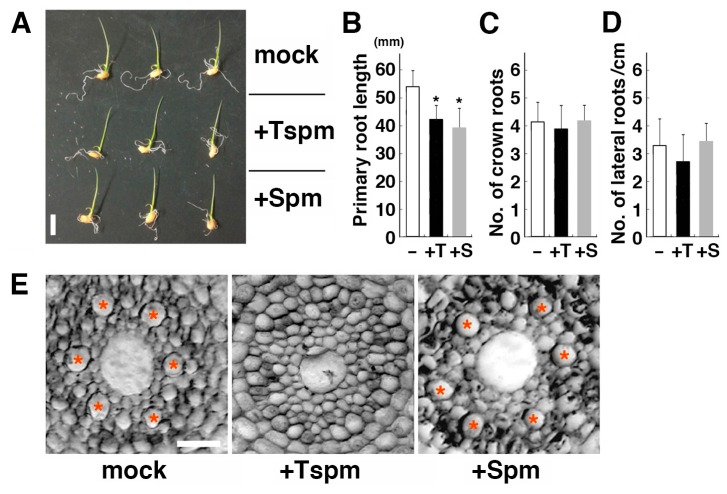
Effect of thermospermine and spermine on the growth of rice seedlings. (**A**) Four-day-old seedlings of rice grown in distilled water with no polyamines (mock), with 50 µM thermospermine (+Tspm), or with 50 µM spermine (+Spm). Scale bar is equivalent to 1 cm. (**B**) Length of the primary root in 4-day-old rice seedlings. (**C**) Number of crown roots in 4-day-old rice seedlings. (**D**) Density of lateral roots per length of axial root in 4-day-old rice seedlings. In (**B**–**D**), seedlings were grown without polyamines (−), 50 µM thermospermine (+T), or 50 µM spermine (+S). Error bars represent SE of five independent experiments with each four seedlings per treatment. Asterisks indicate significantly different values from the control (* *P* < 0.05, Student’s *t*-test). (**E**) Root cross sections prepared from at a 1 cm distance from the root tip of 4-day-old rice seedling. Asterisks indicate xylem vessels with apparent diameter of more than 10 µm. Scale bar is equivalent to 20 µm.

When grown for four days after germination with 50 µM thermospermine, rice seedlings displayed no obvious alteration in the shoot growth compared with those with mock or 50 µM spermine (Figure 2A). However, the length of the primary root was reduced by thermospermine and also by spermine (Figure 2B), indicating that these tetraamines have an inhibitory effect on root elongation in rice. The number of crown roots was not altered by these tetraamines (Figure 2C). In Arabidopsis, exogenous supply of thermospermine severely suppresses differentiation of xylem vessels and also formation of lateral roots [16,34]. In rice, formation of lateral roots was not suppressed by thermospermine (Figure 2D). Microscopic observations of the cross section revealed that rice seedlings grown with thermospermine had no clearly enlarged metaxylem vessels in the root in contrast to those grown with spermine or with no polyamines (Figure 2E) while they showed normal vascular development in leaves (Figure S1).

### 2.2. Thermospermine Reduces OsACL5 Expression but Induces Apoplastic PAO Genes

**Figure 3:**
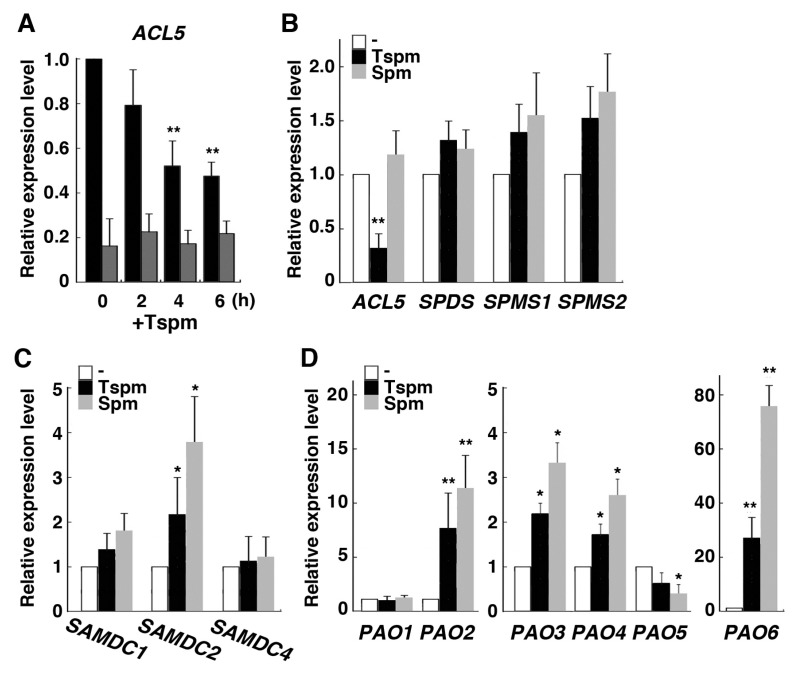
Effect of thermospermine and spermine on polyamine biosynthetic and catabolic genes in rice. (**A**) Time course changes in *OsACL5* expression after treatment of 4-day-old seedlings with 50 µM thermospermine (+Tspm). Black and dark gray bars represent relative expression levels in the root and those in the shoot, respectively. The level in the root before treatment is set as 1. (**B**) Expression levels of *OsACL5*, *OsSPDS*, *OsSPMS1*, and *OsSPMS2*. (**C**) Expression levels of *OsSAMDC* genes. (**D**) Expression levels of *OsPAO* genes. In (**B**–**D**), RNA was extracted from roots after 24-h treatment of 4-day-old seedlings with mock (white bars), 50 µM thermospermine (black bars), and 50 µM spermine (gray bars). The transcript levels in mock treated roots as represented by white bars are set as 1. Error bars represent SE of three independent experiments with each performed in duplicate. Asterisks indicate significantly different values from the control level (* *P <* 0.05, ** P < 0.01, Student’s *t-*test).

We examined whether expression of rice genes involved in polyamine biosynthesis and catabolism are affected by exogenous thermospermine or not. We first examined the time course of the response of *OsACL5* to thermospermine in shoot and root tissues of the seedling and found that *OsACL5* expression was gradually reduced in the root during treatment with 50 µM thermospermine but not altered in the shoot (Figure 3A), suggesting that thermospermine biosynthesis in rice is also under negative feedback control at least in the root. We then focused on the effect of 24-h treatment of the seedlings with thermospermine and spermine on the expression of polyamine-related genes in the root. *OsACL5* was not responsive to spermine and *OsSPDS*, *OsSPMS1*, and *OsSPMS2* also showed no response to thermospermine and spermine (Figure 3B). Expression of *OsSAMDC2* was increased by both thermospermine and spermine, but *OsSAMDC1* and *OsSAMDC4* were not responsive to these polyamines (Figure 3C). Expression of *OsSAMDC3*, a putative ortholog of *AtSAMDC4* involved in thermospermine biosynthesis (Figure 1D), was not detected in the root. On the other hand, expressions of *OsPAO2* and, in particular, *OsPAO6*, both encoding extracellular polyamine oxidases, were drastically increased by both thermospermine and spermine while those of *OsPAO3* and *OsPAO4* were moderately increased by these tetraamines but *OsPAO5* expression was not (Figure 3D). We detected no increase in the expression of *OsPAO1*, a putative ortholog of thermospermine-catabolizing *AtPAO5*, although a previous study has reported on its induction by thermospermine [25]. Expression of *OsPAO7*, which has been shown to be an anther-specific gene [27], was not detected in the root. We confirmed that expression levels of these genes were not altered by thermospermine in the above-ground part of seedlings except for those of *PAO2* and *PAO6*, which were also increased by thermospermine (Figure S2).

### 2.3. Expression of Phloem-Specific Genes are Increased by Thermospermine

**Figure 4:**
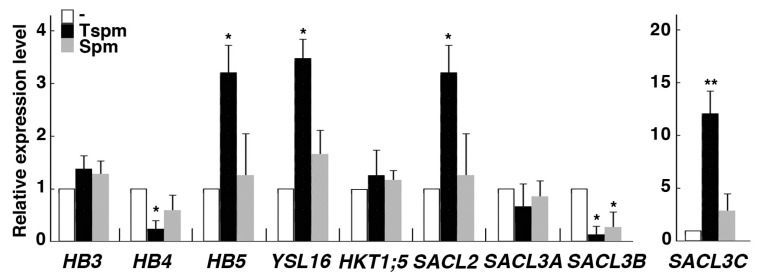
Effect of thermospermine and spermine on the expression of vascular-related genes in rice roots. RNA was extracted from roots sampled 24-h after treatment of 4-day-old seedlings with mock (white bars), 50 µM thermospermine (black bars), and 50 µM spermine (gray bars). The transcript levels in mock treated roots as represented by white bars are set as 1. Error bars represent SE of three independent experiments with each performed in duplicate. Asterisks indicate significantly different values from the level before treatment (* *P <* 0.05, ** P < 0.01, Student’s *t-*test).

In Arabidopsis, thermospermine reduces expression of a number of genes involved in xylem differentiation [34], including all members of the Class III homeodomain leucine zipper (HD-Zip III) gene family, which is known to play a regulatory role in vascular development in Arabidopsis [35]. Then we examined the effect of thermospermine on the expression of vascular-related genes in rice roots. *OsHB3* expression was not altered by 24-h treatment with thermospermine, while *OsHB4* expression was reduced and *OsHB5* expression was increased by the same treatment (Figure 4). *OsHB3* and *OsHB4* are the closest homologs of *PHB* and *PHV* of the Arabidopsis HD-Zip III gene family and phloem-specific *OsHB5* shows the highest similarity to other HD-Zip III members, *ATHB8* and *CNA*, although expression patterns in rice are different from those in Arabidopsis [36]. We also examined expression of OsYSL16, which encodes a copper-nicotianamine transporter localized in phloem [37], and that of OsHKT1;5 encoding a sodium transporter in root xylem parenchymal cells [38]. *OsYSL16* expression was increased in response to thermospermine while *OsHKT1;5* showed no response (Figure 4). Furthermore, four genes belonging to the *SAC51* family [39] were examined. Expression of *OsSACL2* and *OsSACL3C* was markedly increased by 24-h treatment with thermospermine but *OsSACL3B* expression was reduced by thermospermine and spermine (Figure 4). In the shoot part, however, expression levels of these genes were not altered by 24-h treatment with thermospermine (Figure S2).

## 3. Discussion

This study provides an initial investigation of physiological and molecular effects of exogenous thermospermine in rice seedlings. We confirmed that thermospermine represses both development of metaxylem vessels and expression of *OsACL5* in the root, suggesting its functional conservation between eudicots and monocots. It may be concluded that thermospermine, whose biosynthesis is under negative feedback control of the expression of a gene for thermospermine synthase, is involved in the repression of xylem development at least in the root of angiosperms. We observed no apparent effect of thermospermine on leaf vasculature and *OsACL5* expression in the shoot. Optimal concentrations of thermospermine might not be reached at the site of action in the shoot probably because of the presence of apoplastic PAOs. Alternatively, it is also conceivable that in the rice shoot, vascular development is uncoupled from the function of thermospermine. In addition, lateral root formation, which is severely repressed by exogenous thermospermine in Arabidopsis, was not affected in rice. Development of the protoxylem, which plays a role in triggering lateral root initiation, might be less affected in rice than in Arabidopsis under our experimental condition of thermospermine treatment.

In terms of root elongation, both thermospermine and spermine were shown to be inhibitory to the growth. For rice seedlings that are normally grown in water, high concentrations of these polyamines may be generally toxic to the growth. The inhibitory effect of spermine on the growth of rice seedlings has been reported previously [40]. We found that except *OsACL5*, expressions of many polyamine-related genes were more or less increased by both thermospermine and spermine. These include *OsSAMDC2*, *OsPAO2*, *OsPAO3*, *OsPAO4*, and *OsPAO6*. There is accumulating evidence suggesting the importance of PAOs, in particular, apoplastic PAOs in stress responses [41,42,43]. A previous study has shown that expressions of some of rice *PAO* genes are responsive to salt stress [44]. We suggest that exogenous thermospermine and spermine may mimic the stress signal to trigger *OsPAO* gene activation. However, such a drastic increase in the expression of *PAO* genes by spermine or thermospermine has not been reported for *PAOs* in Arabidopsis and only the *AtPAO5* expression has been shown to be increased by high salt [45]. The high toxicity of exogenous polyamines in rice seedlings might be attributed to hydrogen peroxide or other products of apoplastic polyamine degradation. On the other hand, considering the function of SAMDC in providing a substrate for polyamine biosynthesis, induction of *OsSAMDC2* by polyamines seems contradictory. *OsSAMDC2* expression has also been shown to be increased by high salinity [21]. It is thus possible that, in water-grown rice seedlings, exogenous polycationic thermospermine and spermine are sensed as stress to induce stress-responsive genes including *OsSAMDC2*. How expressions of *OsPAO*s and *OsSAMDC2* are induced by polyamines or high salt remains an open question.

Expression of *OsHB5*, which was increased in response to thermospermine, is in contrast to that of *ATHB8* and *CNA* in Arabidopsis, which are reduced by thermospermine [34]. This might be related to different tissue expression patterns of HD-Zip III genes between rice and Arabidopsis. Importantly, expression of phloem-specific *OsYSL16* was also increased by thermospermine. This suggests a possibility that thermospermine is involved not only in repressing xylem development but also in promoting phloem development in rice. So far we have obtained no cytological evidence of the role of thermospermine in phloem development and it should be pursued in future work. Furthermore, among the members of the *SAC51* family, *OsSACL2* and *OsSACL3C* were up-regulated by thermospermine while *SACL3B* was down-regulated. Our recent study has shown that 5’ leader regions of *OsSACL3A* and *OsSACL3C* mRNAs can be responsive to thermospermine in terms of enhancing translation of the downstream reporter in transgenic Arabidopsis [39] while no genes that are transcriptionally and specifically up-regulated by thermospermine have been identified in Arabidopsis. Tissue-specific expression patterns of the rice *SAC51* family genes remain to be investigated.

In conclusion, our results revealed that thermospermine plays a repressive role in xylem vessel development in the root of rice seedlings and it also affects expression of a subset of genes that are different from those reduced in Arabidopsis. This difference might be largely due to the presence of apoplastic PAOs in rice, whose gene expression was found to be strongly induced by both thermospermine and spermine.

## 4. Materials and Methods

### 4.1. Chemicals

Spermine-4HCl and thermospermine-4HCl were obtained from Nakarai Chemicals (Kyoto, Japan) and Santa Cruz Biotechnology (Santa Cruz, CA, USA), respectively.

### 4.2. Plant Material and Growth Conditions

The rice *Oryza sativa* L. cv. Nipponbare was used throughout this work. Seeds were sown in Petri dishes containing distilled water and incubated at 22 °C on an orbital shaker at 60 rpm under 16 h light/8 h dark condition. The dishes were arranged as a single layer in a randomized complete block design. The growth experiments were repeated five times with each four seedlings per treatment.

### 4.3. Preparation of *Sections and Microscopy*

Root tissues were fixed in FAA (45% ethanol, 4% formaldehyde, 5% acetic acid), dehydrated through an ethanol series, and embedded in Technovit 7100 resin (Heraeus Kulzer, Wehrheim, Germany). Samples were sectioned into 10 µm-thick slices by a rotary microtome (RM2245, Leica Microsystems, Wetzlar, Germany) equipped with a tungsten carbide disposable blade (TC65, Leica). Sections were stained with 0.5% Toluidine blue and observed under a differential interference contrast microscope (SMZ-ZT-1, Nikon, Tokyo, Japan).

### 4.4. RNA Preparation and RT-PCR

Total RNA was extracted from tissues with mortar and pestle in the presence of liquid nitrogen and isolated by using NucleoSpin RNA Plant kit (Macherey-Nagel, Düren, Germany) according to the manufacture’s instruction. Each RNA sample was prepared three times independently. The resulting RNA solution was treated with RNase-free DNase I (Takara, Kyoto, Japan). First-strand cDNA was synthesized using PrimeScript II 1st strand cDNA synthesis kit (Takara) with an oligo (dT) primer. Quantitative real-time PCR was performed using KAPA SYBR Fast qPCR kit (Kapa Biosystems, Woburn, MA, USA) with gene-specific primers (Table 1 and Table 2) in a Thermal Cycler Dice TP760 System (Takara). *OsACT1* [46] was used as an internal control. All RT-PCR experiments were done in duplicate using three distinct cDNA preparations per sample.

### 4.5. Phylogenetic Analysis

Deduced protein sequences were aligned by ClustalW and phylogenetic trees were constructed using the neighbor-joining method in the DDBJ website (http://clustalw.ddbj.nig.ac.jp/index.php?lang=ja). The trees were visualized by TreeDyn (http://www.phylogeny.fr/one_task.cgi?task_type=treedyn). Accession numbers of rice genes are listed in Table 1 and those of Arabidopsis genes are AtACL5, At5g19530; *AtSPDS1*, At1g23820; *AtSPDS2*, At1g70310; *AtSPMS*, At5g53120; *AtSAMDC1*, At3g02470; *AtSAMDC2*, At5g15950; *AtSAMDC3*, At3g25570; *AtSAMDC4*, At5g18930; *AtPAO1*, At5g13700; AtPAO2, At2g43020; *AtPAO3*, AT3G59050; *AtPAO4*, AT1G65840; and AtPAO5, AT4G29720.

## Figures and Tables

**Table 1:** List of polyamine biosynthetic and catabolic genes examined in this study and primer sequences used for RT-PCR.

Gene Name	Locus ID	MSU ID	Primer Sequence
*OsSPDS*	Os07g0408700	LOC_Os07g22600	F: CAACATACCCTAGTGGTGTT
			R: CTAGTTGGCCTTGGATCCAA
*OsSPMS1*	Os06g0528600	LOC_Os06g33710	F: CCTGAAGGGAAATATGATGC
			R: AATGACACCACTAGGATAGG
*OsSPMS2*	Os02g0254700	LOC_Os02g15550	F: CGACATATCCCAGTGGTGTG
			R: CAATACGCCTCTAGCTCTCT
*OsACL5*	Os02t0237100	LOC_Os02g14190	F: AAGAGTAGGGAGAAGTTCGA
			R: GTGTATGCTTTGACATACTTGA
*OsSAMDC1*	Os04g0498600	LOC_Os04g42095	F: ACTCCAACTGCGCGAAGAAG
			R: CAGCAGCAGACAAGACACCC
*OsSAMDC2*	Os02g0611200	LOC_Os02g39795	F: GCTTACTCCAACTGCGCGAG
			R: CGCCGAGACCGGTGGAGAGT
*OsSAMDC3*	Os05g0141800	LOC_Os05g04990	F: GTGGTGGACGAGAATGACCC
			R: CTAGTTGTCATGCTCATGCT
*OsSAMDC4*	Os09g0424300	LOC_Os09g25625	F: TGCTTACTCCAACTGCGCTC
			R: CATAGCCTTCAAACCCAATG
*OsPAO1*	Os01g0710200	LOC_Os01g51320	F: TTCCTCGGGTCATACAGCTA
			R: CTACGTGGTGTGATTCGCTC
*OsPAO2*	Os03g0193400	LOC_Os03g09810	F: CCCAGATTCCAATGTTCTTC
			R: GCAGAGTCAATACCTGCAAG
*OsPAO3*	Os04g0623300	LOC_Os04g53190	F: CTGCCGAGCCGATACATTAC
			R: CATCTCCAGCATGTCCAGCT
*OsPAO4*	Os04g0671200	LOC_Os04g57550	F: CCACTGAACCTACGAAGTAT
			R: ATCTCCTCGTAGGCCTTGAC
*OsPAO5*	Os04g0671300	LOC_Os04g57560	F: GCTACTGAACCGGTCCAGTA
			R: GGAAAAGGTCGGAGATGCCT
*OsPAO6*	Os09g0368200	LOC_Os09g20260	F: ACGGAGTCTGGCAGGAGTTT
			R: CGCCCTGAGCTGGTCATAC
*OsPAO7*	Os09g0368500	LOC_Os09g20284	F: ACGGAGTCTGGCAGGAGTTT
			R: CGCCCTGAGCTGGTCATGT

**Table 2:** List of other genes and their primer sequences used for RT-PCR.

Gene Name	Locus ID	MSU ID	Primer Sequence
*OsHB3*	Os12g0612700	LOC_Os12g41860	F: GATCATGCAGCAGGGTTTCA
			R: ATACGGTGGTGGTATTCAGG
*OsHB4*	Os03g0640800	LOC_Os03g43930	F: CTGCTCCCTGAAGGCTGCTC
			R: ATGACCAGTTGACGAACATG
*OsHB5*	Os01g0200300	LOC_Os01g10320	F: CAACATCATGGAGCAGGGGA
			R: TGTACACGCTGTTTCATGAG
*OsYSL16*	Os04g0542800	LOC_Os04g45900	F: CTTAACAACAGAGTGGCGGA
			R: AGAGCGCGATCTTGCCGTAG
*OsHKT1;5*	Os01g0307500	LOC_Os01g20160	F: CGAGGTTATCAGTGCGTATG
			R: GCATGGGTGCTTGCAGTTAG
*OsSACL2*	Os03g0391700	LOC_Os03g27390	F: CCCAAGATTGCCAGGCCGAG
			R: GGATCCCATCAAGAAACAACCA
*OsSACL3A*	Os03g0591300	LOC_Os03g39432	F: GCAAGTGTGCCAGGCCGAAT
			R: AGATCTGGGAAAGCAGGAAATG
*OsSACL3B*	Os02g0315600	LOC_Os02g21090	F: GTCATCGTGTGAGAGCAAG
			R: ACGTCACGGGCTTGAGAAG
*OsSACL3C*	Os01g0626900	LOC_Os01g43680	F: CAAGTGTGCCAGGCTGAGTA
			R: GGATCCTCTACCTGATCTGATG
*OsACT1*	Os03g0718100	LOC_Os03g50885	F: CTCCCCCATGCTATCCTTCG
			R: CCATCAGGAAGCTCGTAGCT

## Supplementary Materials

The following are available online at https://www.mdpi.com/2223-7747/8/8/269/s1, Figure S1: Effect of thermospermine on the leaf vasculature of rice seedlings, Figure S2: Effect of thermospermine on the expression of polyamine- and vascular-related genes in the shoot of 5-day-old seedlings.
