# Supplementary material for: Effect of Thermospermine on the Growth and Expression of Polyamine-Related Genes in Rice Seedlings
Source: Plants (Basel). 2019 Aug 6;8(8):269. doi: 10.3390/plants8080269 (PMC6724145; doi:10.3390/plants8080269)
Supplement: Supplementary file 1 [file plants-08-00269-s001.pdf]

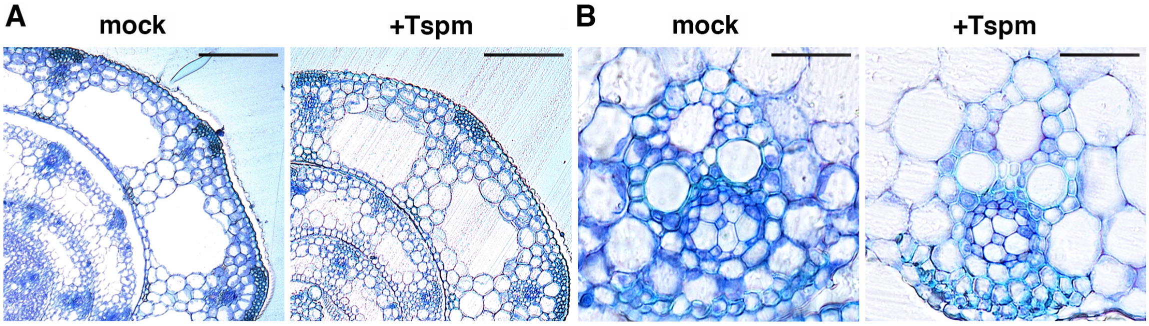

Figure S1. Effect of thermospermine on the leaf vasculature of rice seedlings.
(A) Leaf cross sections of 4-day-old rice seedling grown in distilled water with no polyamines (mock) and with 50 μM thermospermine (+Tspm). Scale bars are equivalent to 200 μm. (B) Higher magnification of the sections of leaf vasculature of rice seedling grown as in (A). Scale bars are equivalent to 50 μm.

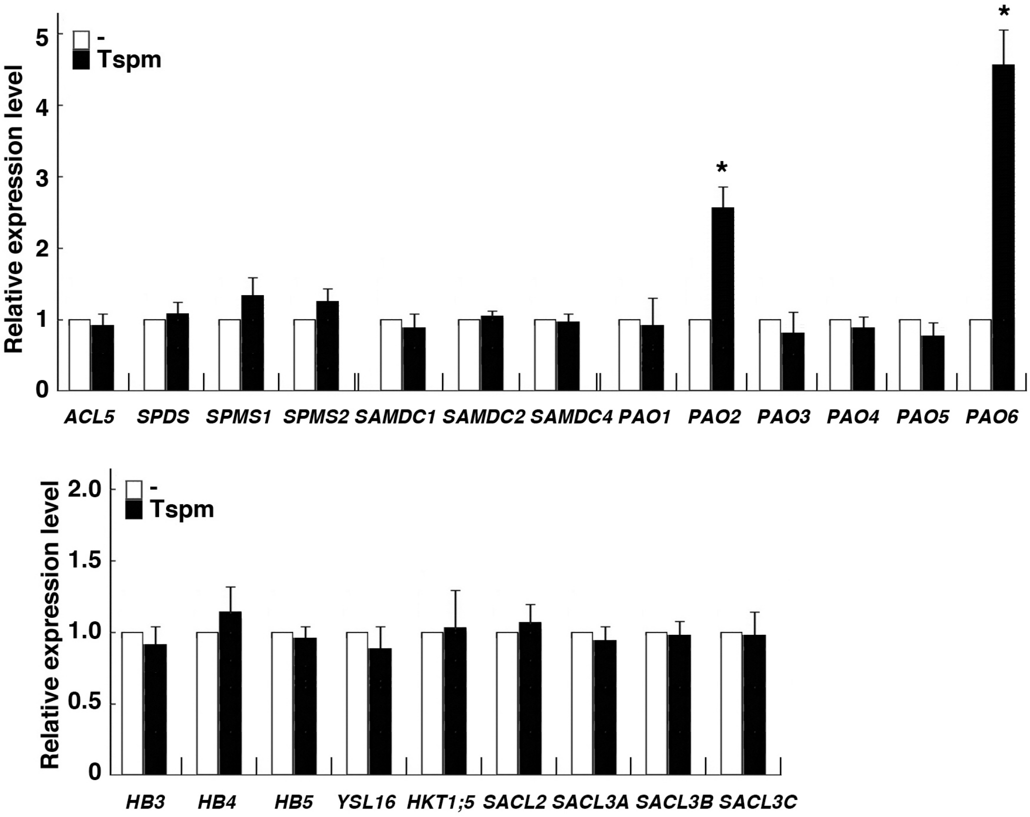

Figure S2. Effect of thermospermine on the expression of polyamine- and vascular-related genes in the shoot of 5-day-old seedlings. RNA was extracted from the above-ground part of 4-day-old seedlings treated with mock (white bars) and 50 μM thermospermine (black bars) for 24h. The transcript levels in mock as represented by white bars are set as 1. Error bars represent SE of three independent experiments with each performed in duplicate. Asterisks indicate significantly different values from the control level (**P* < 0.05, Student's *t*-test).
